# Hind limb motoneurons activity during fictive locomotion or scratching induced by pinna stimulation, serotonin, or glutamic acid in brain cortex‐ablated cats

**DOI:** 10.14814/phy2.13458

**Published:** 2017-09-28

**Authors:** Sergio H. Duenas‐Jimenez, Luis Castillo Hernandez, Braniff de la Torre Valdovinos, Gerardo Mendizabal Ruiz, Judith M. Duenas Jimenez, Viviana Ramirez Abundis, Irene Guadalupe Aguilar Garcia

**Affiliations:** ^1^ Department of Neurosciences CUCS Universidad de Guadalajara Guadalajara Jalisco Mexico; ^2^ Basic Center Department of Physiology and Pharmacology Universidad Autónoma de Aguascalientes Aguascalientes Mexico; ^3^ Department of Computational Sciences CUCEI Universidad de Guadalajara Guadalajara Jalisco Mexico; ^4^ Department of Physiology CUCS Universidad de Guadalajara Guadalajara Jalisco Mexico

**Keywords:** Glutamic acid, locomotion, scratching, serotonin

## Abstract

In brain cortex‐ablated cats (BCAC), hind limb motoneurons activity patterns were studied during fictive locomotion (FL) or fictive scratching (FS) induced by pinna stimulation. In order to study motoneurons excitability: heteronymous monosynaptic reflex (HeMR), intracellular recording, and individual Ia afferent fiber antidromic activity (AA) were analyzed. The intraspinal cord microinjections of serotonin or glutamic acid effects were made to study their influence in FL or FS. During FS, HeMR amplitude in extensor and bifunctional motoneurons increased prior to or during the respective electroneurogram (ENG). In soleus (SOL) motoneurons were reduced during the scratch cycle (SC). AA in medial gastrocnemius (MG) Ia afferent individual fibers of L6‐L7 dorsal roots did not occur during FS. Flexor digitorum longus (FDL) and MG motoneurons fired with doublets during the FS bursting activity, motoneuron membrane potential from some posterior biceps (PB) motoneurons exhibits a depolarization in relation to the PB (ENG). It changed to a locomotor drive potential in relation to one of the double ENG, PB bursts. In FDL and semitendinosus (ST) motoneurons, the membrane potential was depolarized during FS, but it did not change during FL. Glutamic acid injected in the L3‐L4 spinal cord segment favored the transition from FS to FL. During FL, glutamic acid produces a duration increase of extensors ENGs. Serotonin increases the ENG amplitude in extensor motoneurons, as well as the duration of scratching episodes. It did not change the SC duration. Segregation and motoneurons excitability could be regulated by the rhythmic generator and the pattern generator of the central pattern generator.

## Introduction

The episodes of real or fictive locomotion occurring in precolicular decerebrated cats (Bayev 1978; Perret and Cabelguen 1980) are initiated by the locomotor regions of the brainstem (Orlovsky 1972; Mori et al. 1978; Garcia‐Rill et al. 1985; Ryczko and Dubuc 2013), particularly by the mesencephalic locomotor region (MLR) activating via segmental spinal interneurons liberating excitatory amino acids (Douglas et al. 1993). These episodes preserve many of the characteristics of normal locomotion (even in the absence of motor cortex) as a consequence of a regulatory action of vestibular and thalamic reticular nuclei, as well as the contribution of the sensory feedback (Beloozerova and Sirota 1993a,b; Hiebert and Pearson 1999; Arshian et al. 2014; Marlinski and Beloozerova 2014). During fictive locomotion in immobilized cats, the sensory input is absent, although most of the locomotion pattern is preserved using only spinal cord central pattern generator (CPG).

The spinal cord CPG for locomotion seems to be constituted by neural elements placed on two levels: one producing the rhythm for FL or FS and the other producing several motor patterns in a hind limb extensor and flexor motoneurons (McCrea and Rybak 2007). These levels are related to interneurons producing reflexes and timing recruitment in the motoneurons (Prochazka et al. 2002), but with phase‐locking asymmetries at flexor–extensor transitions during fictive locomotion (Boothe et al. 2013).

Descending synaptic inputs segregate the scratching or stepping motor programs because of their synaptic effects in the pathways fibers converging in the CPG neurons (Sirois et al. 2013). Therefore, it is important to study whether hind limb individual motoneurons are segregated to make adequate tasks with a different duty cycle and a different muscle force generation (Shimanskii and Baev 1986; Kuhta and Smith 1990). Thus, it is of interest to assess whether SOL motoneurons innervating a slow muscle were active during the scratching fast duty cycle in BCAC.

The segmental peripheral inputs, differing from the supraspinal control, serve basically to mold steps and segmental limb trajectories, although CPGs can produce the basic motor pattern for different tasks (Armstrong 1988; Shen and Poppele 1995; McCrea and Rybak 2008). However, afferent inputs act to control the CPG for adequate load (Grillner and Lund 1968), step patterns, and balance (Mori et al. 1978; Musienko et al. 2012, 2014). Therefore, it is also important to study hind limb FS and FL to value the CPG activity in the absence of afferent inputs in BCAC. During these motor behaviors, a task‐dependent activity with electromyography patterns consistent with muscle fiber type could be observed in extensor muscles (Hodson‐Tole et al. 2012). A task dependency also occurred in presynaptic inhibition. For example, the AA reduction during scratch was attributable to a task dependency in transmission in primary afferent depolarization (PAD) pathways and not to underlying potential oscillations related to the CPG (Cote and Gossard 2003). In thalamic cats, HeMR during fictive locomotion has already studied in extensor muscles (Duenas and Rudomin 1988). However, these reflexes are not fully analyzed during FS in BCAC. Therefore, it is of interest to study the HeMR produced in SOL, FDL, flexor hallucis longus (FHL), PB, motoneurons, innervating a slow extensor, and bifunctional muscles. It is also of interest to analyze motoneuron excitability by motoneuron intracellular recording and whether AA in Ia afferent fibers is modulating the HeMR during FS. There is evidence for specialized rhythm‐generating mechanisms in mammalian spinal cord. Data indicate that cyclic period, phase durations, and phase transitions are not regulated similarly during fictive locomotion or scratching (Frigon and Gossard 2010; Gossard et al. 2011). However, in precolicular decerebrated cats, when a compulsory activity took locomotor place following an scratching episode, flexor, extensor, and intermediate single interneurons rhythmically fired in the same phase during both the scratching and subsequent postscratching locomotion. The fact that no changes in the phases of these neurons from scratching to postscratching locomotion were found suggests that in the lumbar spinal cord there are neurons associated with both motor tasks (Trejo et al. 2015). It would be interesting to observe whether postscratching locomotion also occurs in BCAC cats and to analyze whether bifunctional motoneurons are segregated for FL or FS.

Locomotion activity is favored by dopamine, serotonin, and glutamic acid agonist drugs. In chronic spinal cats, 5‐hydroxytrytophan, a precursor of serotonin, and serotonin agonist drugs, 5,5‐methoxy‐N, N, N‐dimethyltryptamine and quapazine, increase the step length and the amplitude of extensor and flexors, as well as axial muscles, and lowered the threshold to produce cutaneous reflexes (Barbeau and Rossignol 1987). Glutamic agonist drugs also facilitated locomotion in vivo and in vitro preparations (Cazalets et al. 1996). In immobilized BCAC, the serotonin or glutamic acid effects when injected in the L3‐L4 spinal cord level are not known. Therefore, this issue was also studied since pinna stimulation could generate FS or FL using different neurotransmitters modifying the rhythm generator or the patterns generator forming the spinal CPG (Lafreniere‐Roula and McCrea 2005). These authors discussed that the maintenance of cycle period timing during some deletions suggests a separation of the functions of rhythm generation and the distribution of excitation to motoneurons in the organization of locomotor and scratch CPGs. They found that deletions occur in a qualitatively homogeneous way throughout the motor pool. They also show that deletions were either accompanied by a reduction or the complete absence of the expected motoneurons depolarization. On the other hand, even small decreases in premotoneuronal drive could result in substantial loss of motoneuron recruitment in those motoneurons depolarized to levels just threshold for voltage‐depended excitation. They found that deletions with and without alterations in cycle period were found in both scratch and locomotion. Thus, the common characteristics of deletions suggest that there may be a similar organization of the networks responsible for fictive locomotion and scratch.

Thus, it is of interest to study whether pinna stimulation activates the rhythmic and the pattern generator modules embedded in the CPG using different neurotransmitters. It is also important to assess whether a presynaptic mechanism acting in proprioceptive Ia afferent fibers segregates hind limb motoneurons changing the heteronymous motoneuron recruitment for adequate FL or FS tasks.

## Material and Methods

### General procedures

All procedures were performed in accordance with the ethical considerations guidelines of the Mexican Official Norm (NOM‐062‐ZOO‐1999) and the National Institutes of Health Guide NIH, Publication No. 8023 (1996) for the Care and Use of Laboratory Animals. In addition, the experimental protocols were approved by the Institutional Bioethical Committee of the Institutional Animal Care and Use Committee (IACUC).

A total of 16 cats (11 males and 5 females) were used in these experiments. Immobilized BCAC with a mass of 3–3.5 kg each were used to study fictive locomotion and scratching. All animals were raised in separate cages. The brain cortex ablation was performed under ketamine (20 mg/kg) and brevital (20–40 mg/kg). It was made by brain cortex and suprathalamic structure ablation, as well as some thalamic nuclei (Lopez Ruiz et al. 2017).

### Fictive locomotion and scratching

In immobilized BCAC, fictive scratching or locomotion was evoked by light pressure in the pinna, once a topical application of d‐tubocurarine (0.01 to 0.025%) on the C1‐C2 spinal cord segments had been made. Immobilized cats were maintained by artificial ventilation adjusted to sustain the expiratory CO_2_ level from 4% to 5%.

### Electroneurograms recording

In all cats studied, a computer with an analog to digital converter was used to digitize the analog ENG signal from two to eight nerves (A Digi Data(1200) series interface and the axoscope 7 computational program were used). Two AM System differential AC amplifiers (four channels by amplifier) Model 1700 were used for ENG signal amplification. When records were made in two nerves, 10 kHz sampling was used, 8 kHz sampling was used when four channels were recorded, and 4 kHz when records were made in eight nerves. Sometimes, the ENG activities were rectified and averaged. In some cases, an autocorrelation or cross‐correlation among ENGs was calculated. This analysis was performed based on the following algorithm:

* £

* A(I,K)*A(J,K+d)

* £

where K = d, I,J are the ENG channels to be correlated, d indicates the displacement in time between channels I and J. The interval goes from ‐* £ to £ *. The elements of the chosen sample are A (I,K). The program formulated by this algorithm graphically exhibits the normalized autocorrelation and the cross‐correlation between two channels. This algorithm was based on Person and Mishin (1964), but the correlations were normalized to the maximal value in order to plot the graphs. In some locomotion or scratching episodes, the ENG activity was recorded in single units in FDL or MG nerves. The single unit was isolated after selective sectioning and splitting the nerves with a tungsten wire. In a thin nerve filament, a sorting by a computer program from other motor units was made. This was made by a computation algorithm performed in our laboratory allowing the unit sorting by three parameters: (1) spike amplitude, (2) peak to peak spike amplitude, and (3) spike duration. The instantaneous firing frequency (IFF) of these single units was analyzed and plotted. When we studied the HeMR, three to four ENG were simultaneously recorded using the AC differential amplifiers, one of them containing HeMR and two containing motor activity in different hind limb nerves. A reference pulse signaling time stimulus was also recorded, and all four signals were stored in an FM tape for further analysis. ENGs were digitized (8 kHz), HeMR (peak to peak voltage amplitude wave) was measured for all responses occurring in a scratching episode.

The time length of each cycle was normalized and subdivided into 10 bins to increase temporal resolution of the statistical analysis (Alaburda et al. 2005). The mean and standard deviation of all the peak to peak HeMR amplitude values corresponding to each bin of the pre‐ and during bursting periods were computed and evaluated for statistical differences using a one‐way ANOVA at a significance level of *α *= 0.05 with one degree of freedom adjusted with a Bonferroni correction for multiple comparisons (*α *= 0.005, *n* = 10) (Welch and Ting 2009). Figure 3B–D depicts the obtained HeMR mean (circles) and standard deviation (vertical lines) values for each bin.

The additional circle and vertical line at the left of the ordinate corresponds to the statistics of the prior to scratching episodes. The stars indicate the bins for which there were statistically significant differences. All scratching cycles not differing more than 15% from the mean cycle duration were chosen to evaluate the average scratching cycle duration (continuous line). In cases when ENG activity in SOL was absent, a black and white bar at the top of the graph was plotted. In these cases, we used FHL for separating extensor and flexor cycle phases (Fig. 3E). Additionally, the SC, phase extensor, and flexor durations were evaluated and plotted as horizontal bars below each graph.

Note that the central tendency of the first bin is always higher for the HeMR values depicted in Figure 3B–D. In Figure 3B, more than 50% of the values of those depicted have a statistically significant higher value with low dispersion of the data. Figure 3C presents two higher means with larger dispersions and one lower value. Figure 3D presents two high values and five with lower values, all of them with a small variance. Finally for Figure 3E, all the bins present statistically significant lower values than the prescratching period.

### Antidromic activity in single Ia afferent fibers

**Figure 1:**
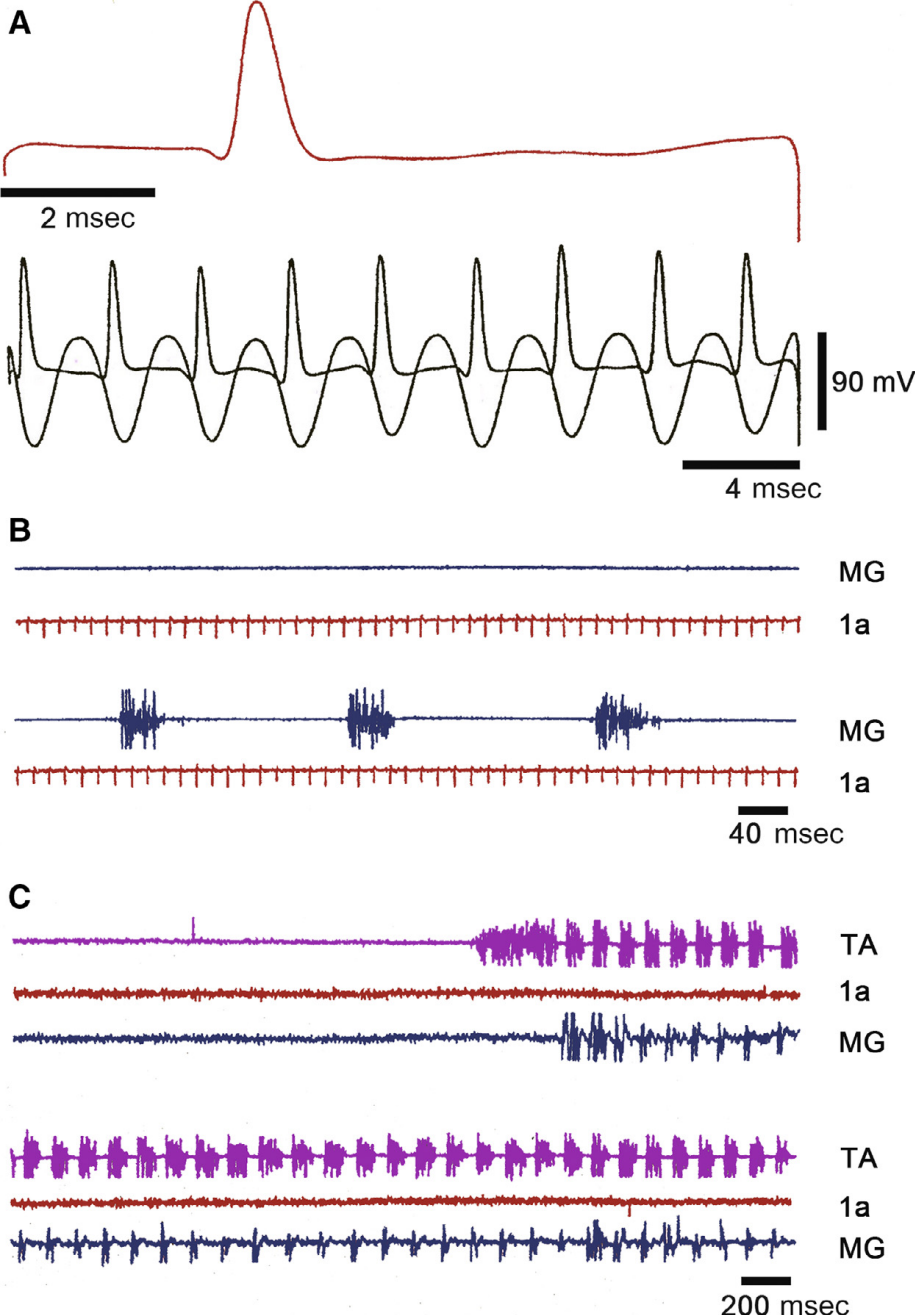
Orthodromic action potentials in a single 1a afferent fiber of a dorsal root filament produced by MG nerve electrical stimulation or by MG muscle stretching. (A) Orthodromic action potential recorded in a single1a MG afferent fiber in a L6 dorsal root, first trace. (B) Orthodromic action potentials in the same 1a afferent fiber (second trace) produced by MG muscle high‐frequency muscle stretchings (third trace). (B) Upper two traces were made prior to fictive scratching; lower two traces records made during fictive scratching, trace as indicated. (C) Absence of antidromic action potentials in the 1a afferent fiber. Traces as indicated. Lower three traces are consecutives of the upper three traces. The records were taken after a cut of the single 1a afferent fiber distant from the dorsal root entry zone.

A L6‐L7 dorsal root filament was dissected in six different cats and divided until obtaining a single afferent fiber. The filament was placed on an electrode for differential amplification. We identified and recorded Ia afferent fiber in L6‐L7 dorsal roots when action potential in the single afferent fiber is produced by electrical stimulation of the MG nerve (Fig. 1A). It has a conduction velocity faster than 100 m/sec. This single dissected afferent can follow gastrocnemius muscle stretches over 200 Hz (Fig. 1B). A total of 17 single afferent fibers were studied during rest and during FS (Fig. 1C). AA was value when a single Ia afferent fiber in the L6 or L7 dorsal root was cut far away from its dorsal root entry zone (Fig. 1).

The motoneuron activity was recorded intracellularly in 62 motoneurons in 10 cats. However, motoneurons were recorded only in three FDL, three PB, two ST, and three MG when scratching episodes were followed by fictive locomotion episodes or in ST motoneurons when FL was followed by FS. Motoneuron intracellular recordings were made with glass micropipettes filled with potassium acetate 2.5 mol/L.

### Analysis of fictive scratching–locomotion phenomena in BCAC microinjected with serotonin or glutamic acid in spinal cord

An L3‐L4 intraspinal microinjection of serotonin (0.5 mL, 7 *μ*mol/L, 2 *μ*L/min) was applied in three BCAC. Glutamic acid (1 ml, 10 *μ*mol/L, 1 *μ*L/min) was injected in L3‐L4 spinal cord region in four BCAC to evaluate their effects on hind limb nerve extensor and flexor ENGs during FL or FS. In two BCAC cats, we also investigated the effects of glutamic acid spinal cord microinjection when these cats had spontaneous FL.

## Results

### Fictive locomotion and scratching

**Figure 2:**
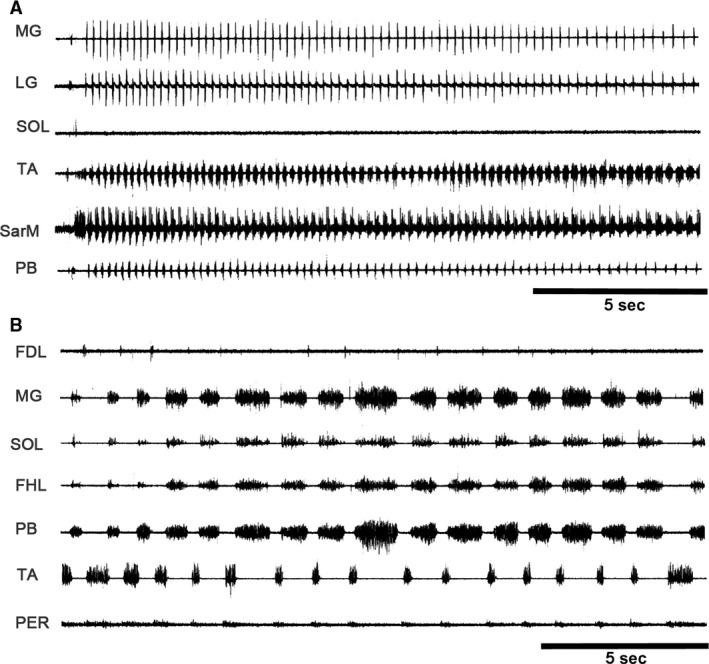
Pinna mechanical stimulation produces fictive scratching or fictive locomotion (A) Hind limb fictive scratching in several nerves produced by pinna stimulation. Traces as indicated. Note a short TA approach period duration during the scratching episode. During fictive scratching SOL ENG appeared without activity, traces as indicated. (B) A well‐defined SOL ENG activity during fictive locomotion.

In three immobilized cats with left extended hind limbs and right flexed hind limbs, pinna stimulation produced episodes of fictive scratching followed by episodes of fictive locomotion. They occurred in only 3 of 10 cats. Figure 2 exhibits a period of scratching (Fig. 2A), and subsequently, a fictive locomotion episode (Fig. 2B). The rhythm transition occurred only when the scratching approach period in flexor muscle as TA was reduced or absent (Fig. 2A). Notice an apparent absence of SOL ENG during FS.

### Heteronymous monosynaptic reflex in thalamic scratching cats

**Figure 3:**
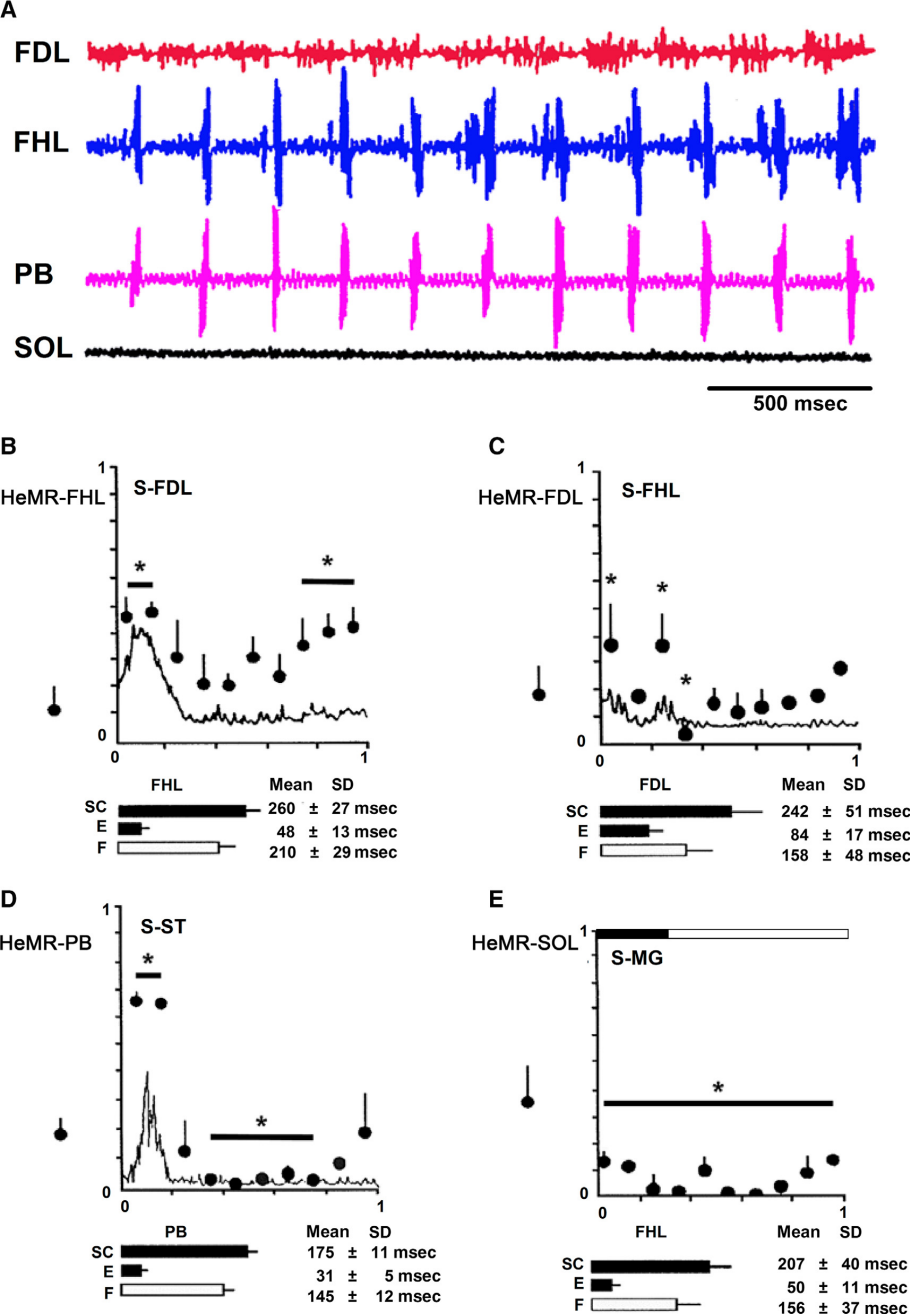
Heteronymous monosynaptic reflex modulation during scratching in thalamic cats during fictive scratching. (A) Scratching episodes in a thalamic cat. Flexor digitorum longus (FDL), flexor hallucis longus (FHL), posterior biceps (PB), and soleus (SOL) electroneurograms, one to four traces, respectively. (B) HeMR amplitude changes in FHL motoneurons (ordinate), produced by electrical FDL nerve stimulation (S‐FDL) plotted in relation to normalized FHL scratch cycle (abscissa). Upper bar below the abscissa (SC) denotes cycle duration as measured in the FHL electroneurogram. Second and third bars below the abscissa are the extensor and flexor phase durations. The dot with the vertical line on the left of the ordinate indicates the HetMR amplitude and standard deviation before the scratching episode. (C) HeMR amplitude changes in FDL motoneurons in relation to normalized FDL scratch cycle. Electrical stimulation was given to the FHL nerve. (D) PB‐HeMR amplitude changes (ordinate) in relation to the PB scratch cycle (abscissa). Electrical stimulation was given in ST nerve (1.2 × T). The continuous line in (B–D) indicates the averaged and rectified FHL, FDL, and PB ENGs of the respective scratch cycles. (E) Amplitude changes in SOL‐HeMR (ordinate) during scratch cycle (abscissa). The normalized phases of all scratching episodes cycles are indicated by the horizontal upper bar plotted on the graph. The extensor phase is indicated in black and the flexor phase, in white. The mean scratching cycle duration and its standard deviation (SD) were measured in the FHL electroneurogram which is an extensor synergist. (B–E) Horizontal bars below the abscissa indicate mean duration and the line indicates SD. D: Asterisk in A–D denotes statistical differences of *P* < 0.05.

Hind limb scratching motoneuron patterns exhibit the absence of activity in SOL electroneurograms (5 cats, 15 scratching episodes). FHL motoneurons exhibited cyclic ENG out of phase with the FDL motoneuron activity, but in phase with PB ENG (Fig. 3A). FDL HeMR amplitude increased during and before FHL ENG (Fig. 3B).

**Figure 4:**
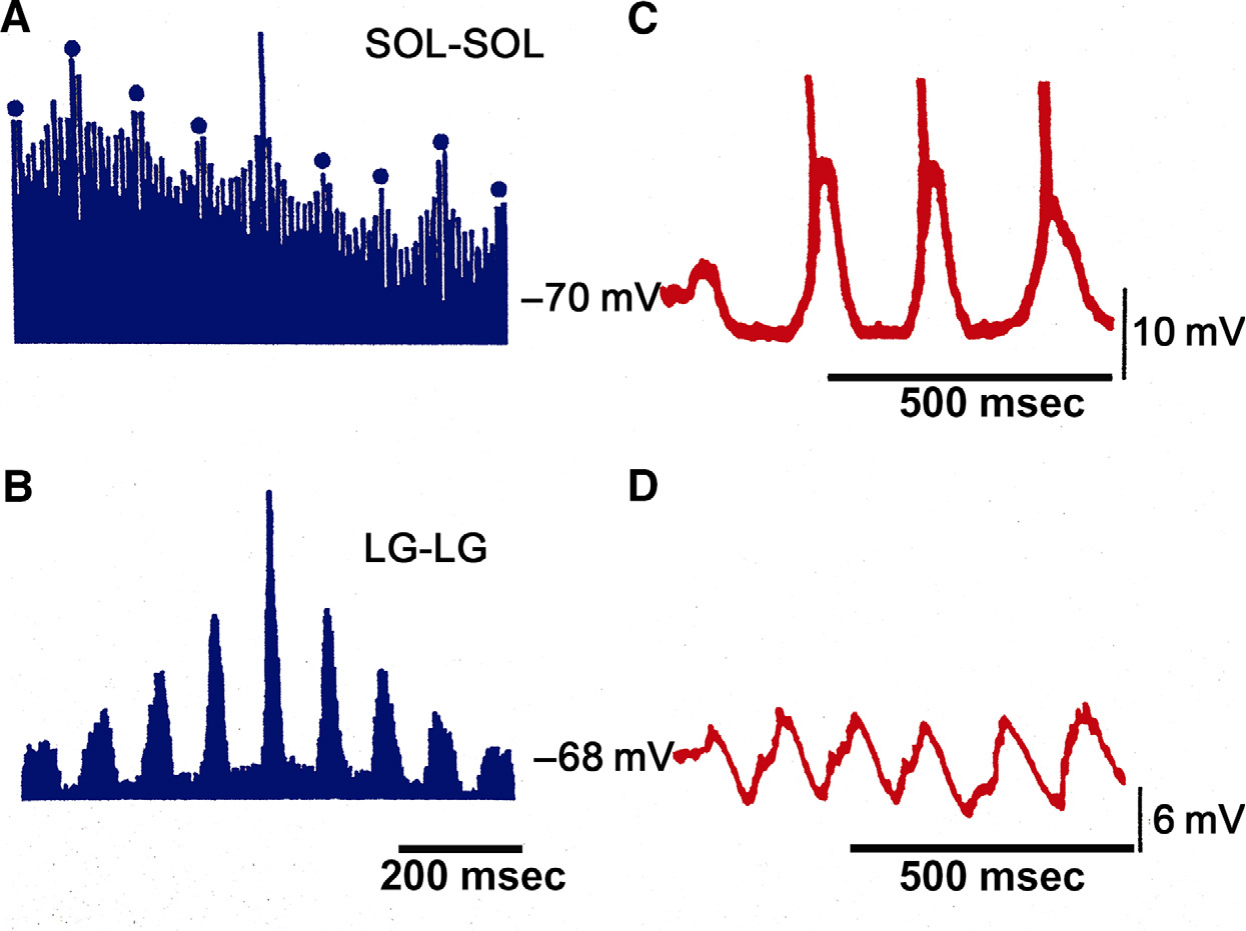
Autocorrelation analysis in LG and SOL ENG and intracellular recording in SOL motoneurons. (B) Autocorrelation of the electroneurogram recorded in a LG nerve filament (LG‐LG). (A) Exhibits the autocorrelation analysis in the SOL electroneurogram (SOL‐SOL). Both autocorrelations were taken in the same scratching episode. The graph was normalized to the maximal correlation value. Dots in A denote autocorrelation increases in the SOL electroneurogram. Note a coincidence in the autocorrelation between LG and SOL peaks. (C) Membrane potentials changes in a SOL motoneuron during FS. (D) Membrane potential changes in other SOL motoneurons in a different FS episode.

FDL and PB HeMR increase in phase with respective ENG. An amplitude reduction was observed between the HeMR amplitude increases. A HeMR amplitude reduction in FDL and PB occurred between the fourth and the sixth scratching cycle bins. The HeMR amplitude was reduced to 78–100% of its amplitude recorded prior to scratching episodes. In BCAC, the SOL heteronymous monosynaptic reflex was reduced throughout and after the scratching episodes (from 1 to 10 sec). This reduction varied from 60% to 98% of its value taken prior to scratching activity (Fig. 3E). Interestingly, some of the SOL neurons recorded in the SOL nerve exhibit rhythmic activity firing. It was observed when the SOL ENG was autocorrelated. This activity was in phase with LG nerve activity (Fig. 4A and B). Intracellular recording of SOL motoneurons (*n* = 4) exhibits a sustained membrane potential hyperpolarization. In one of the four neurons, the scratching drive potential was large enough to produce action potentials (Fig. 4C). In three other neurons, the sustained hyperpolarization was large enough to prevent action potential generation (Fig. 4D). During scratching, excitability of motoneurons seems to be the main factor for modulating HeMR, since a decrease in PAD occurred during fictive scratching (Sirois et al. 2013). However, AA occurs in cutaneous afferent fibers during FS (Gossard et al. 1989). They could possibly appear in MG Ia afferent fibers. Their generation could be occurring at any place, from the dorsal root entry zone to the synaptic contact of Ia afferent fibers with motoneurons or interneurons. This could be an alternative presynaptic mechanism modulating the SOL, HeMR. To study this point, we recorded action potentials in single Ia afferents in L6‐L7 dorsal roots. A total of 17 Ia individual afferent fibers of MG before and during FS were recorded. In the absence of FS, we stretched the MG muscle and recorded Ia afferent action potentials in dorsal root filaments. In all cases, stretching the MG muscle produces orthodromic action potentials at or near 100 Hz. This frequency was similar during FS. Then, we distally cut the single Ia afferent fiber to the dorsal root entry zone, none of the 17 Ia afferent fibers exhibited AA during FS (Fig. 1C).

### Motoneuron firing during FS

**Figure 5:**
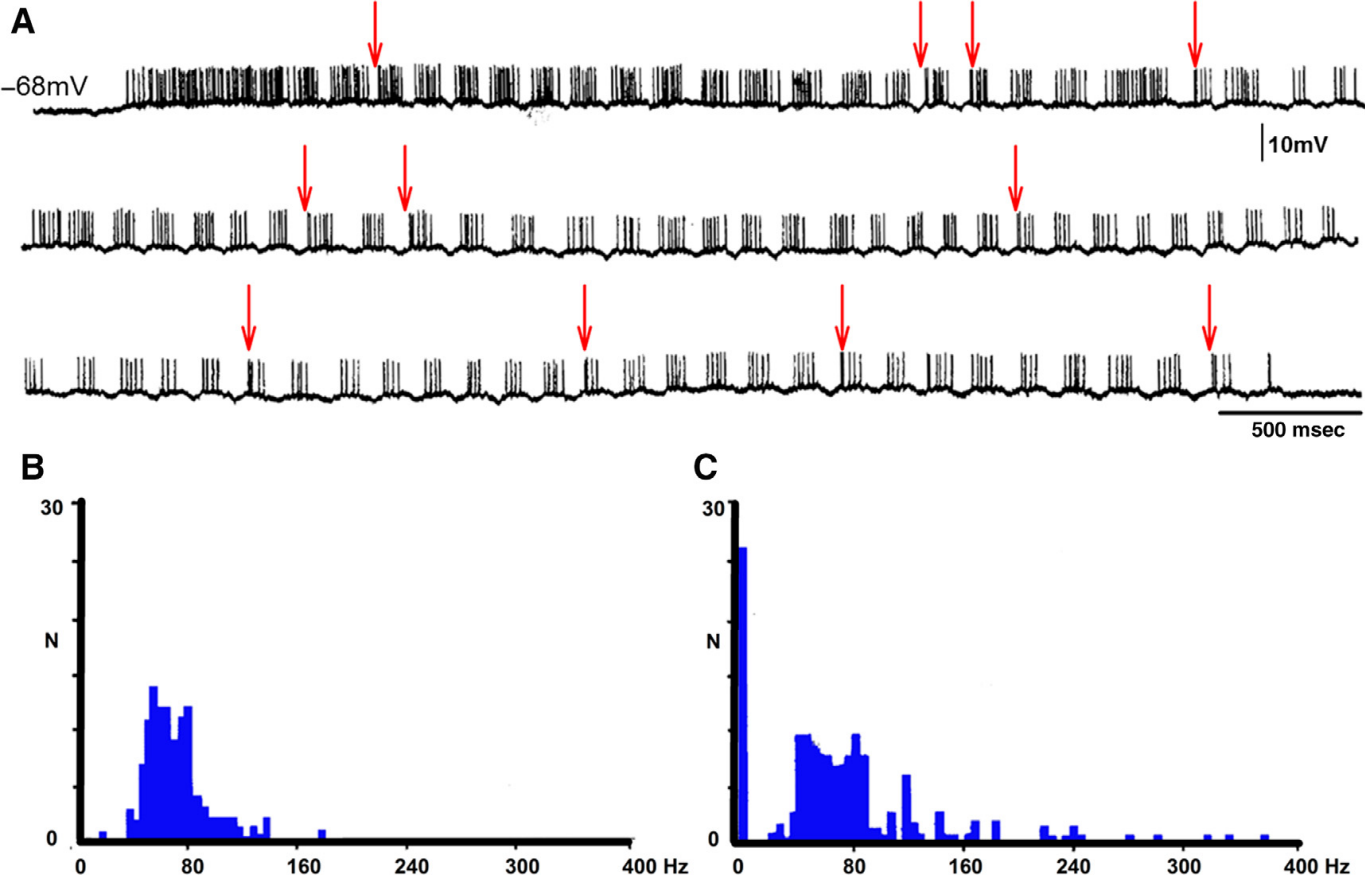
Intracellular recording in a FDL motoneuron and in a FDL nerve filament during fictive scratching. (A) Resting potential changes and spiking activity in a flexor digitorum longus (FDL) motoneuron during a scratching episode. Note the initial sustained depolarization (scratching approach period) in a FDL motoneuron, as indicated by a reduction in the resting potential, followed by cycling depolarizations. Also, note the doublets (arrows) at the onset of several FDL motoneurons spiking bursts. (B) Histogram showing the instantaneous firing frequency during the period of initial tonic activity during a scratching episode recorded in a FDL nerve filament. (C) Histogram of the instantaneous firing frequency during the cyclic (rhythmic) period recorded in the FDL nerve filament in the same scratching episode. The vertical (4–6 Hz) denotes the inverse of the interval between scratching motor bursts (1/t).

To study motoneuron firing, either a thin filament of FDL and MG nerves was used or an intracellular recording in FDL, PB, ST, and MG motoneurons was made. In FDL motoneurons (*n* = 2), IFF varied from 30 to 150 Hz during the initial short duration tonic activity (approach period, Fig. 5B). During the rhythmic period, FDL firing frequency ranged from 30 to 350 Hz (Fig. 5C). Frequencies over 150 Hz did not appear during the approach period (Fig. 5B). The intracellular records of FDL motoneurons allowed detecting motoneuron spikes at the onset of the rhythmic driving potential cycles with a very high frequency (Fig. 5A) which are denominated doublets in decerebrate cats (Zajac and Young 1980).

**Figure 6:**
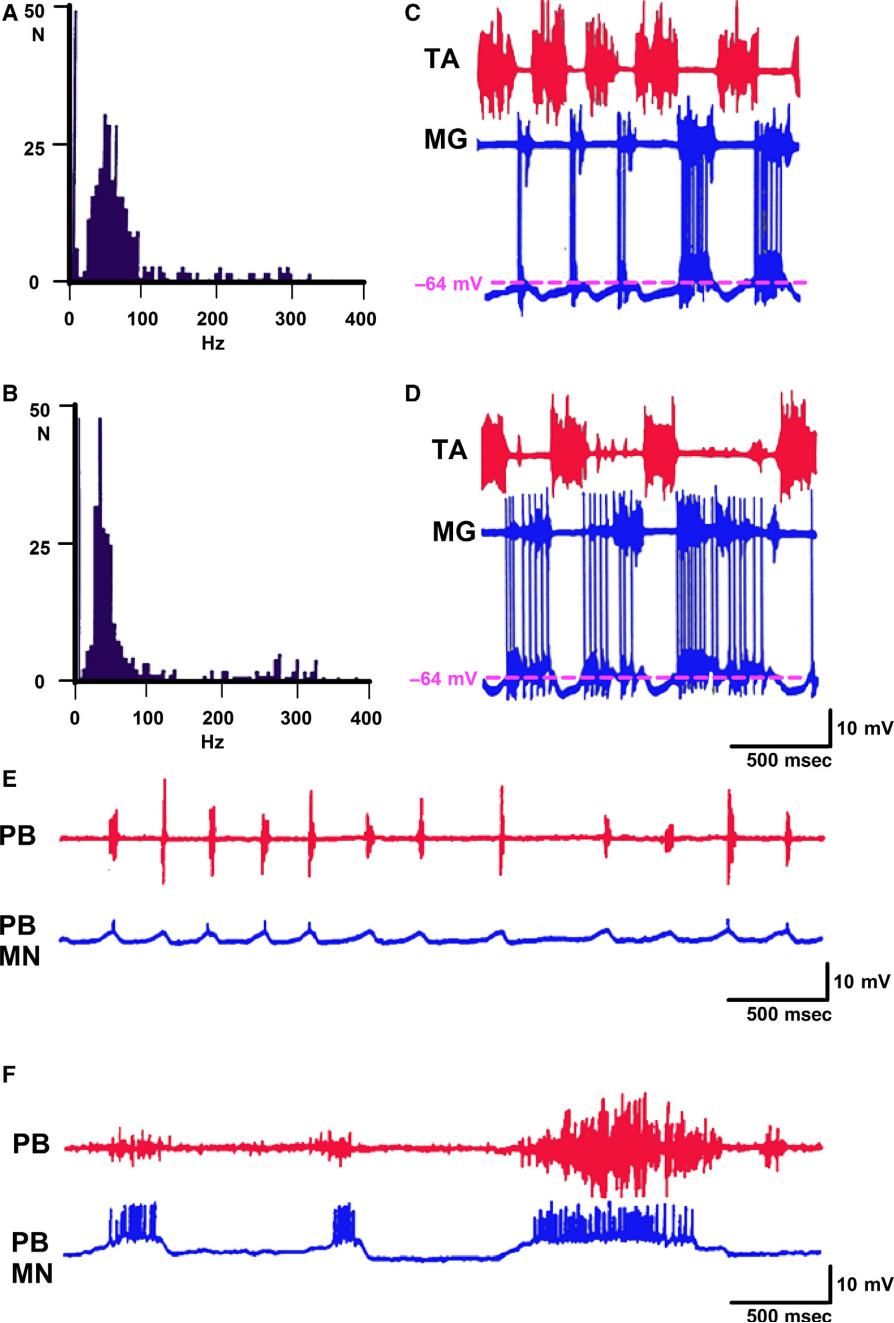
Instantaneous firing frequency in a single unit recorded in a medial gastrocnemius (MG) nerve filament and intracellular recording in MG, and PB motoneurons during scratching followed by locomotion. (A, B) Ordinate: number scratching cycles or fictive locomotion cycles, respectively. Abscissa: inverse of intervals between spikes. (A) The instantaneous firing frequency illustrated in the bins one to three, correspond to scratching burst frequencies. (B) The first bin is denoting the inverse of the interval between locomotion motor bursts. Firing activity was recorded in a peripheral MG nerve filament. (C, D) First and second traces: TA, MG ENGs, respectively, third trace, membrane potential in a MG motoneuron. (C) Transition for scratching to locomotion, traces as indicated. (D) Fictive locomotion. C and D are consecutive traces. (E, F) An episode of scratching in a PB motoneuron, followed by fictive locomotion. (E) First trace, electroneurogram in PB nerve illustrating fictive scratching. Second trace, membrane potential changes in a PB motoneurons during scratching. (F) FL locomotion after scratching traces in E and F illustrated the membrane potential changes (second trace) in a posterior biceps motoneuron. First trace ENG in a PB nerve filament during fictive scratching (E) and during fictive locomotion (F). Note the absence of the PB motoneurons membrane potential changes during the short ENG PB locomotor burst.

In MG motoneurons, a presence of high‐frequency firing during rhythmic fictive scratching occurred. Motoneuron firing was recorded in the MG nerve filaments. During locomotion, the most common IFF was 35 Hz (Fig. 6B), whereas during scratching cycles, it was 45 Hz (Fig. 6A). IFF over 150 Hz was found during FL and FS. These results strongly suggest that the same individual MG motoneurons were recruited during locomotion and scratching, although there were some changes in the firing pattern.

**Figure 7:**
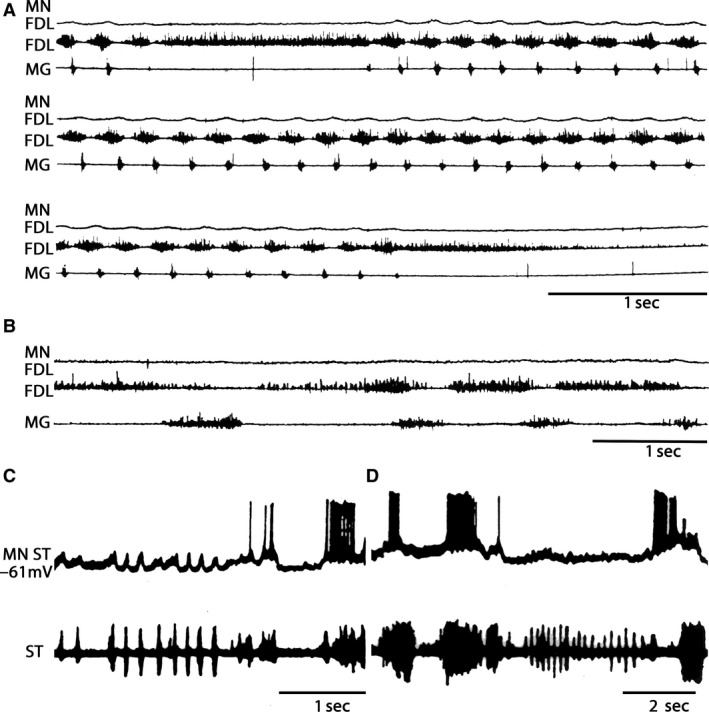
Membrane potential changes in a FDL motoneurons and two ST motoneurons during FL or FS. (A) FDL motoneuron exhibiting phasic depolarizations during FS. Phasic depolarizations were not observed during fictive locomotion (B). Phasic depolarizations in a ST motoneuron, during fictive locomotion or scratching. (C) Phasic depolarization occurred during FL, but not during FS, in other ST motoneurons, in a different cat.

We also recorded membrane potential changes during FS or FL episodes in two MG motoneurons recorded intracellularly. They received synaptic driving potentials, both during FL and fictive FS. Doublets occurred both during FL and FS (Fig. 6C and D). In these MG motoneurons, there was no motoneuron segregation for these two motor tasks. During fictive scratching, PB motoneurons activity presented a single burst related to the scratching driving potential (Fig. 6E). During locomotion, PB motoneurons firing occurred in relation to a long ENG burst, but it was not observed (*n* = 2) during the second short PB ENG burst (Fig. 6F). FDL motoneurons were rhythmically depolarized during FS (Fig. 7A), but not during FL (Fig. 7B). One ST motononeuron was rhythmically depolarized, both during FL and FS (Fig. 7C). In another ST, cyclic motoneuron depolarization was observed only during FL (Fig. 7D). These results suggest ST motoneuron segregation during the FL or FS.

### Serotonin and glutamic acid injected in spinal cord

**Figure 8:**
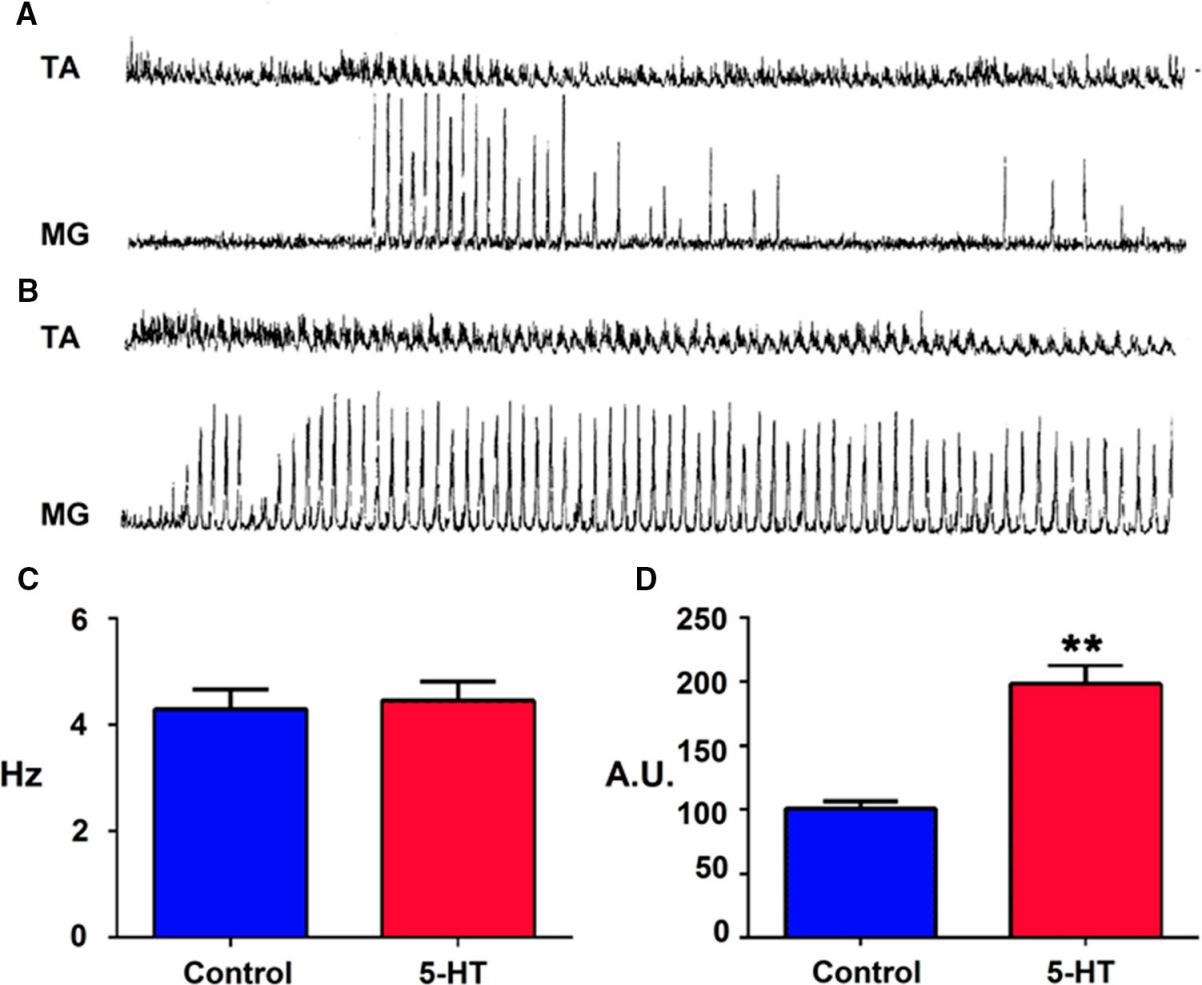
Serotonin effects in the ENG extensor and flexors during fictive scratching. Scratching episodes produced by pinna stimulation before (A) and after serotonin injected in spinal cord (B). Note a duration increase in the scratching episode. (C) Bar graph plotting bursting frequency of 10 scratching episodes before serotonin (control) and after serotonin (5HT, 5 scratching episodes). (D) Bar graph illustrating summed MG burst amplitude before (control, 10, scratching episodes) and after (5HT) spinal cord microinjection (5 scratching episodes), mean (bar), and standard deviation (line) obtained from the episodes of fictive scratching in two cats.

Agonist or antagonist drugs of serotonin or glutamate applied on the lumbar spinal cord modified the fictive patterns (Rossignol et al. 2001, 2004). In the present results, the scratching episodes duration increased after serotonin microinjection (Fig. 8A and B). This result was observed in three cats. Serotonin increased the extensor burst amplitude (Fig. 8D), but their bursting frequency did not change (Fig. 8C). Figure 8D illustrates a graph of the burst amplitude changes measured in 10 scratching episodes before serotonin and five episodes after 5HT microinjection. The rhythmic bursting frequency was similar before and after serotonin microinjection (Fig. 8C). In one of these cats, fictive locomotion was produced by footpad stimulation. MG motoneurons burst amplitude increased, although locomotion frequency did not change (Fig. 10A and B). Note the presence of a deletion in MG ENG, although the MG ENG amplitude increased.

**Figure 9:**
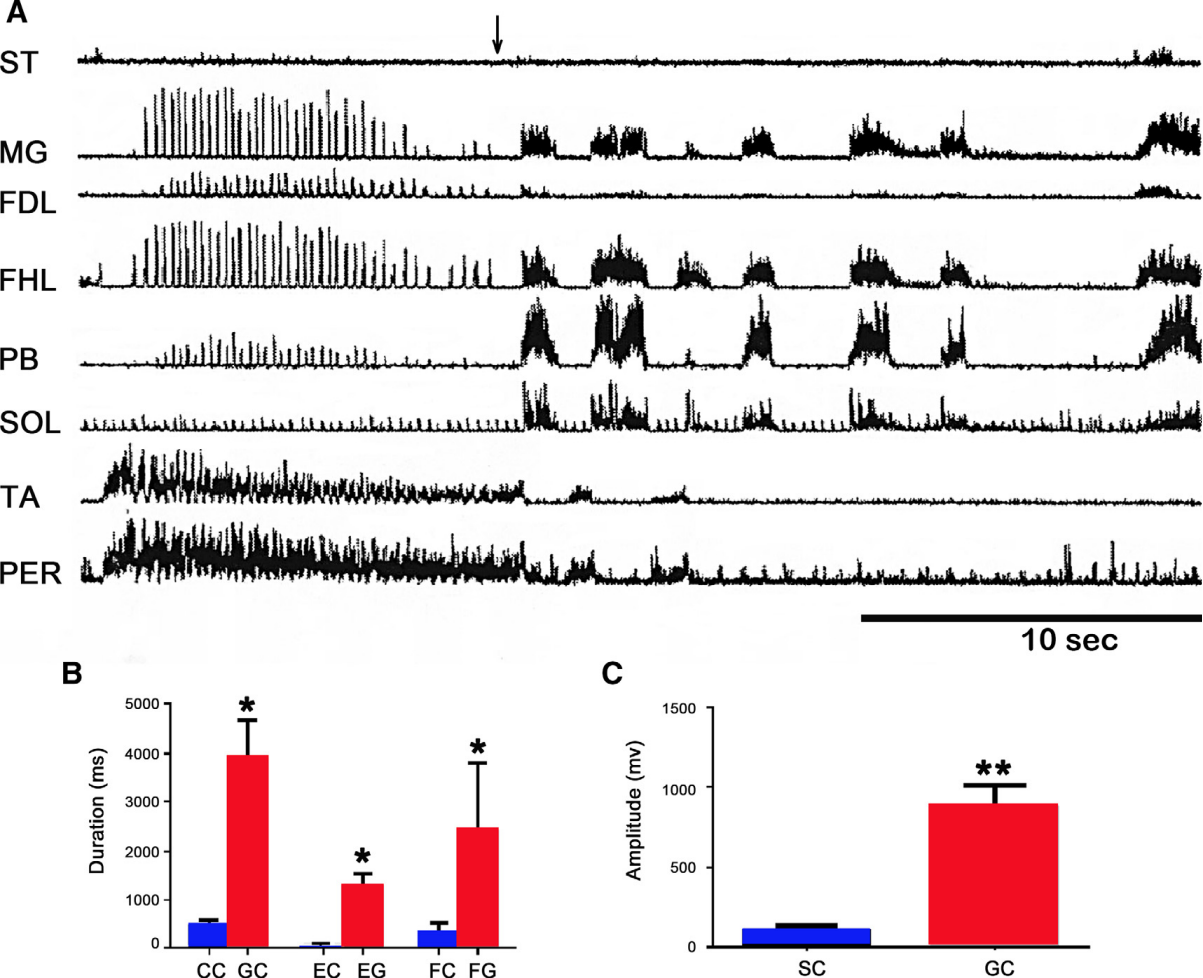
Scratching episode followed by a fictive locomotion episode produced by glutamic acid intraspinal microinjection at L3 spinal cord level. (A) Scratching episode in several nerves, traces as indicated. Note an absence of ENG during scratching in SOL nerve. The scratching episode was followed by fictive locomotion. (B) Scratching cycle duration (CC). Locomotion cycle duration after glutamic acid microinjection (GC). Scratch extensor phase (EC), prior to glutamic acid microinjection. Locomotion extensor phase, after glutamic acid microinjection (EG). Flexor scratching phase (FC) prior to glutamic acid microinjection. (C) SC, summed MG cycle burst amplitude during the scratching episodes. Summed cycle burst amplitude after glutamic acid microinjection (GC) (*n* = 2). A similar scratching and locomotion episode duration was chosen for the amplitude evaluation.

**Figure 10:**
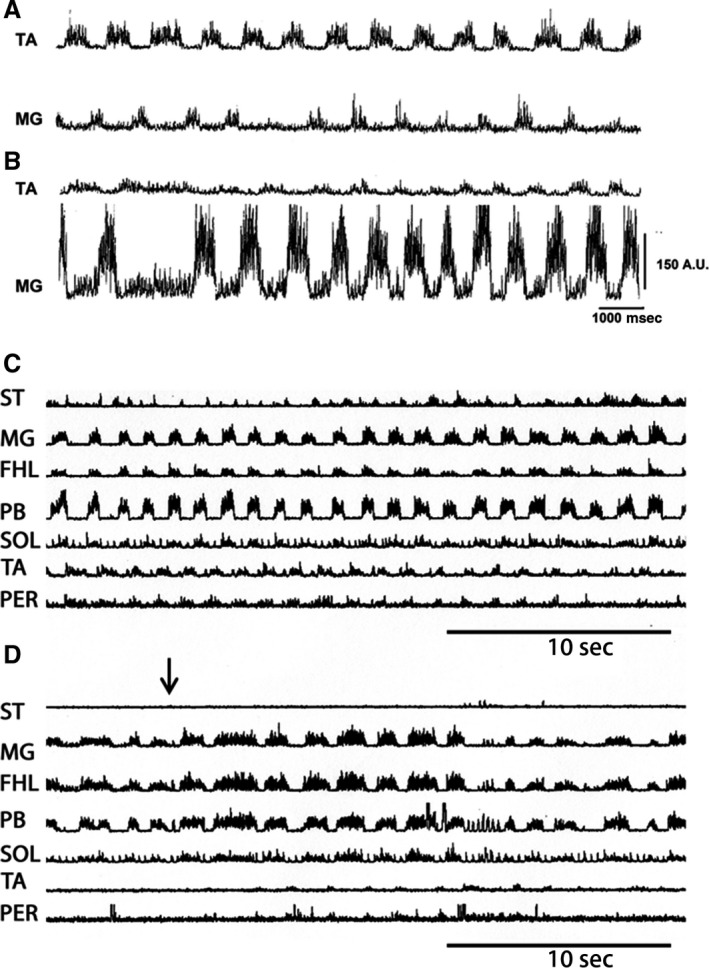
(A) Fictive locomotion produced by footpad stimulation before (upper trace two traces) and after serotonin (lowers two traces). (B) Traces as indicated. (C) Episode spontaneous FL prior to glutamic acid injection (D) ENGs in the same cat after glutamic acid microinjection. Note a duration increase in several extensor ENGs. Also note an amplitude reduction in ST, TA, and PER ENGs. Arrow indicating the onset of glutamic acid injection.

Glutamic acid spinal cord microinjection changed fictive scratching to fictive locomotion (Fig. 9A). The cycle burst duration increased, as well as the extensor and flexor locomotion phases in all studied ENGs. The burst amplitude and duration were measured in the MG ENG (Fig. 9B and C). In two different cats with spontaneous FL, the glutamic acid intraspinal cord microinjection increased the extensor nerve ENG duration, but its amplitude was reduced in flexor ENGs. Figure 10C exhibits spontaneous FL in several nerves in a BCAC. Figure 10D also exhibits the FL episodes in the same cat after glutamic acid spinal cord microinjection. Note deletions in ST, TA, and Per ENG during the duration increase of extensor ENGs.

## Discussion

In immobilized BCAC, we found episodes of FS and FL by pinna stimulation. The pathways producing these tasks should be elucidated in further experiments. HeMR increases during and prior to the respective ENG activity; indicating a motoneurons depolarization in bifunctional motoneurons, prior to the respective ENGs. This can change the excitability in HeMR and change the patterns of motoneurons activity for FS (Lafreniere‐Roula and McCrea 2005). The reduction in SOL HeMR amplitude can be related to a reduction in SOL motoneuron excitability maybe by a prolonged deletion produced by the rhythmic or pattern generator of the spinal cord CPG or by descending pathways acting directly in SOL motoneurons without changing the excitability in the rhythm generator (RG) and the pattern generator (PG) network modules.

AA in MG Ia afferent fibers did not appear during FS, in contrast to those found in cutaneous afferent fibers. Thus, a presynaptic mechanism in MG motoneurons did not contribute to avoid recruitment of SOL motoneurons. SOL, PB, FDL, and ST motoneurons are segregated during FL or FS may be to perform adequate movements. The effects of serotonin and glutamic acid intraspinal microinjection are different; serotonin produced extensors ENG burst amplitude increase, although some deletions remain in the MG ENG. Thus, whereas some motoneurons have an abrupt increase in excitability (increase in ENG burst amplitude), others are silenced maybe by PG network. Glutamic acid changed the motor pattern from FS to FL and increases extensor ENG duration. This could be due to a glutamic action in the RG of the spinal CPG. An increase in extensor ENG duration perhaps contributes to increase force during FL in BCAC.

### Fictive locomotion and scratching

In BCAC cats, topical application of d‐tubocurarine on cervical spinal cord segments and pinna stimulation produces scratching or locomotion in hind limb motoneuron pools. A brain stem–spinal cord pathway seems to activate scratching and locomotion CPG rhythmic generator. However, the scratching generator has neurons inducing the scratching approach period, which did not occur in FL. CPG interneuron activation for locomotion or scratching occurred during the presynaptic inhibition phenomena (Gossard et al. 2011). Lafreniere‐Roula and McCrea (2005) found that except PBSt, the phase of activity of motoneurons pools did not change from fictive locomotion to fictive scratching. We found in ST changes from FL locomotion to FS. This finding occurred in BCAC. In our experiments, only a small number of deletions occurred in serotonin‐ or glutamic acid‐treated cats making a difficult comparison with untreated cats. We did not study reversible or unreversible phase changes after deletions. Therefore, it is difficult to consider the scratch and locomotor networks to be similar.

A maintenance of cycle period during deletions in serotonin‐treated cats suggests that the failure to recruit motoneurons is not inextricably linked to the generation of the locomotor or scratch rhythm. Considering a spontaneous alteration of excitability within the PF network does not affect the RG network as described in McCrea and Rybak (2008). In glutamic acid results changing FS to FL results of a disturbance that affects the RG network. A disturbance at the RG level may directly affect RG for an arbitrary time period. Excitability changes in the RG network would result in spontaneous changes in the cycle period and, depending on the interactions between the RG and PF networks, might not result in deletions of motoneuron activity. We believe that the model developed by McCrea and Rybak (2008) as well as those made by Perez et al. (2009) could explain many of the characteristics found in the present experiments. Although no recordings were made from contralateral nerves, we do not discount the possibility that rhythm generation in the contralateral limb could help maintain ipsilateral cycle period timing during deletions of locomotor activity. Contralateral rhythm maintenance is not likely during fictive scratch because rhythmic activity is very strong in the ipsilateral limb and much weaker in the contralateral limb (Perreault et al. 1999). The present results also favored the idea that PF controls activity in functionally relevant groups of motoneurons (not necessarily those acting at one joint). They also support the unique rhythm generator within the limb. An organization that included strong propriospinal connections to ensure that rhythm generation was synchronized into a single functional entity would be consistent with the absence of AA in Ia afferent fibers. To summarize, the present data are evidence for an organization of the locomotor CPG in which there is a separation of rhythm generation and the distribution of excitation to motoneurons.

### SOL heteronymous monosynaptic reflex regulation by motoneurons excitability changes

During fictive scratching episodes, absence of SOL ENG activity could be attributed to a sustained SOL motoneuron hyperpolarization produced by descending inputs. SOL activity was severely reduced in BCAC but not in spinal cats (not illustrated). The absence of a corticospinal pathway generating extensor motoneuron activity (Leblond et al. 2001; Beloozerova et al. 2006) could be participating in these SOL motoneuron hyperpolarization (Arshavsky et al. 1985; Beloozerova et al. 2006).

In BCAC, SOL‐HeMR was reduced when SOL electroneurographic activity remained silent. However, some SOL motoneurons produced firing and others received subthreshold scratching drive potentials. SOL motoneurons were under some brainstem inhibitory action, since rhythmic activity occurred in most of them during the spinal status (not illustrated). The stimulation of the dorsal portion of the caudal tegmental field evoked membrane hyperpolarization of SOL motoneurons for 40 sec (Arshavsky et al. 1985; Sakamoto et al. 1986). Caudal tegmental field spinal cord pathways seem to possibly participate in the long‐lasting tonic SOL motoneurons inhibition during the scratching episodes in BCAC (Sakamoto et al. 1986). Caution should be taken to attribute a reduction in spinal cord RG or PG excitability networks as producing the long‐lasting SOL deletion. Synaptic effects of descending pathways affecting specific motoneurons without altering RG or PG networks could also contribute to prolonged SOL inhibition.

Brainstem–spinal cord synaptic connection could be used to inhibit slow SOL motoneuron activity during scratching and thereby prevent a mechanical conflict by fast acting muscles. Thus, most SOL motoneurons should be used during fictive locomotion, as occurred in BCAC.

For the rhythmic scratching activity, spinal interneural components, such as commissural interneurons, coordinate the muscle activity (Jankowska 2008) and can be regulated by cerebral cortex, for example, for muscle force production (Lopez Ruiz et al. 2017). FDL and MG motoneurons exhibited during the cyclic period firing frequency ranged from 30 to 350 Hz. Intracellular records of the FDL and MG motoneurons allowed detecting motoneuron spikes (doublets) during rhythmic scratching at the onset of several scratching cycles. The doublets could be indicating an increase in force production during the FDL and MG rhythmic bursts. Doublets could increase muscle force production possibly for readjusting efferent activity, in response to stimulation of muscle afferents and limb position (Shimanskii and Baev 1986, 1987). They may also contribute to increase force generation during locomotion by muscle catch property. However, the force generation in hind limb muscles is reduced in BCAC during locomotion (Lopez Ruiz et al. 2017). An increase of synaptic input in these motoneurons, in the BCAC preparation, could be the cause of doublets or a suppression of slow after hyperpolarization, as observed in turtle spinal neurons during scratching‐like activity (Alaburda et al. 2005). The origin of the descending pathway implied in doublet generation may be localized in thalamic or subthalamic structures, as doublets were also seen in decerebrated cats during fictive locomotion, although spike frequency adaptation did not occur during this task (Brownstone et al. 2011).

### Motoneuron segregation

In the adult cat, CPG express components of particular behaviors leading to the segregation of motoneurons in the same pool. Thus, it seems possible that the CPGs for locomotion and scratching recruited at least two types of PB motoneurons. Indeed, segregation of PB muscle activity had been reported for different motor tasks (Pratt et al. 1991). Posterior biceps motoneuron pools usually exhibit a burst per step cycle only during the early flexion phase (Degtyarenko et al. 1998). Defining the terms of the type of cat preparation for making acceptable validations among different results seems to be of interest. Collectively, in bifunctional muscles, such as PB, FDL, and ST, our results suggest a distributed input in motoneurons by a different set of interneurons, one of them receiving preferential input from the locomotion or the scratching generator. However, MG motoneurons have intermingled inputs of both generators, as occurs in some spinal interneurons (Trejo et al. 2015).

In two MG motoneurons, scratching as well as locomotion generators produced an elevated rate of motoneurons firing, as recorded in MG nerve filaments. This result strongly suggests that the same individual MG unit recruitment occurred during locomotion or scratching, although with some small changes in the firing pattern in both tasks. These firing changes may be produced by a different membrane potential depolarization level in the same motoneurons during FL or during FS (Brownstone et al. 1994).

The presence of HeMR amplitude increase prior to respective ENG activity could be due to motoneuron depolarization favoring motoneurons firing by the excitatory synaptic input of stimulated nerve Ia afferent fibers. When we tested this, the stimuli from heteronymous ST nerve evoked monosynaptic firing in PB motoneurons prior to the ENG activity (not illustrated). This issue deserves to be analyzed in several FDL, FHL, and PB motoneurons in further experiments.

### Serotonin and glutamic acid during the FS–FL phenomena

An increase of serotonin in spinal cord and the activated serotonin receptors in laminae VII and VIII interneurons (Noga et al. 2009) could be important for increasing muscle extensor activity in locomotion. It could also be important during scratching or other motor tasks, even after a mortoneuron hyperpolarization, as occurred in SOL motoneurons (Krawitz et al. 2001). The generation of dendritic potential as those elicited in motoneurons by caproamide effects could be producing action potentials, even in hyperpolarized motoneurons (Duenas et al. 1994).

After serotonin microinjection, the hind paw mechanical stimulation induces higher ENG burst in extensor motoneurons, compared to nonserotonin‐treated cats. It may have been caused by activating the pattern formation network of extensors of the ankle and hip with the inverse consequence for the flexor one.

The change of scratching to locomotion in mesencephalic cats walking on a treadmill has been reported by Mori et al. (1978). It also occurred in immobilized premammilar–precollicular decerebrated cats (Trejo et al. 2015) and in the BCAC. FL episodes are favored by glutamic acid spinal cord microinjection. Glutamic acid could be favorable to the presence of locomotion episodes by activating NMDA receptors or indirectly by activating noradrenergic receptors (Cazalets et al. 1996; Rossignol et al. 1998, 2001; Marcoux and Rossignol 2000; Giroux et al. 2003).

A systemic intracellular analysis of a large number of motoneurons must be studied for comparing motoneuron excitability changes during scratching, versus fictive locomotion changes in BCAC, particularly deletions in bifunctional motoneurons during the transition period.

## Conflict of Interest

No conflicts of interest, financial or otherwise, are declared by the authors.
